# Exploring the Dimensions of Pre-Clinical Research: 3D Cultures as an Investigative Model of Cardiac Fibrosis in Chagas Disease

**DOI:** 10.3390/biomedicines12071410

**Published:** 2024-06-25

**Authors:** Clara Monteiro Seydel, Beatriz Matheus de Souza Gonzaga, Laura Lacerda Coelho, Luciana Ribeiro Garzoni

**Affiliations:** Laboratório de Inovações em Terapias, Ensino e Bioprodutos, Instituto Oswaldo Cruz, Fundação Oswaldo Cruz, Rio de Janeiro 21040-360, Brazil; seydelclara@gmail.com (C.M.S.); biagonzaga04@hotmail.com (B.M.d.S.G.); llacerdac@gmail.com (L.L.C.)

**Keywords:** three-dimensional cell culture 1, cardiac spheroids 2, Chagas disease 3, therapy 4

## Abstract

A three-dimensional (3D) cell culture can more precisely mimic tissues architecture and functionality, being a promising alternative model to study disease pathophysiology and drug screening. Chagas disease (CD) is a neglected parasitosis that affects 7 million people worldwide. *Trypanosoma cruzi’s* (*T. cruzi*) mechanisms of invasion/persistence continue to be elucidated. Benznidazole (BZ) and Nifurtimox (NF) are trypanocidal drugs with few effects on the clinical manifestations of the chronic disease. Chronic Chagas cardiomyopathy (CCC) is the main manifestation of CD due to its frequency and severity. The development of fibrosis and hypertrophy in cardiac tissue can lead to heart failure and sudden death. Thus, there is an urgent need for novel therapeutic options. Our group has more than fifteen years of expertise using 3D primary cardiac cell cultures, being the first to reproduce fibrosis and hypertrophy induced by *T. cruzi* infection in vitro. These primary cardiac spheroids exhibit morphological and functional characteristics that are similar to heart tissue, making them an interesting model for studying CD cardiac fibrosis. Here, we aim to demonstrate that our primary cardiac spheroids are great preclinical models which can be used to develop new insights into CD cardiac fibrosis, presenting advances already achieved in the field, including disease modeling and drug screening.

## 1. Introduction

Over the past decades, advances in cell culture methods have enabled the development of three-dimensional (3D) cell cultures as spheroid models. Using this type of cell culture, some benefits can be observed, for example, (i) cells have more space to grow and interact; (ii) the structure avoids cell flattening; (iii) it improves signals transduction, genic expression, cell proliferation, differentiation and tissue organization; and (iv) it allows for a uniform distribution of adhesion molecules and receptors in cell surface [1,2]. The structure of these microtissues varies according to the methodology used. For example, it can be scaffold-based or not, in which different materials can be used, adding more complexity to the model and enhancing the specific behaviors and morphology of cells. All these factors contribute to creating more robust experiments, which more reliably mimic the microenvironment of an in vivo tissue than two-dimensional (2D) cell cultures [3,4].

There are a few techniques capable of producing spheroids, providing a diversity of 3D culture systems, from the simplest to the most complex. Hanging drop is based on using surface tension and gravitational force to produce the microtissues, being a scaffold-free system [5,6]. Liquid overlay is also a scaffold-free system, which uses nonadhesive materials to inhibit cellular adhesion [7,8]. Pellet culture is based on concentrating cells using centrifugal force, while spinner culture, a similar process, forms cellular aggregates by continuous stirring. Rotating wall vessels are based on constant circular rotations, recreating microgravity to generate the microtissues. Magnetic levitation is based on using a magnetic structure to form the spheroids. Finally, some methodologies represent progress in the field, like microfluidics: an advanced platform in which a microfluidic flow and permeable materials are used to dilute factors [9,10,11,12]. This allows survival and proliferation to be improved, providing a personalized microenvironment that permits co-cultures, creating a dynamic system that mimics the tissues structure and function even more, enabling different kinds of analysis [13]. 

Once microtissues can be cultured using a variety of cell types and techniques, they present many advantages and have versatile applications. This includes the fact that they are great models for analyzing host–pathogen interactions. This is due to their microenvironment, that permits the pathogenic agent and the host to interact in ways that resemble the in vivo environment, but using a much more controlled environment than animal models [14,15]. Therefore, Chagas disease is a neglected tropical illness, caused by the protozoan *Trypanosoma cruzi*, which leads to nearly 12.000 deaths worldwide each year [16]. It is a multisystemic disorder, comprising an acute and a chronic phase [17,18]. The acute phase is mostly asymptomatic, but patients can present severe symptoms such as heart failure and sudden death, resulting in 50.000 deaths being related to cardiac manifestations in CD per year, according to the World Health Organization (WHO) [19,20].

In the chronic phase, the systemic inflammatory process caused by *T. cruzi* infection can lead to important clinical manifestations, such as megaesophagus and megacolon, which represent the digestive form of the disease, as well as some neurological involvement and cardiac effects [21,22,23]. The cardiac form of CD is the most common manifestation, affecting 20–30% of chronic patients [24,25]. The pathophysiology of chronic Chagas cardiomyopathy (CCC) has been discussed in many studies [26,27]. Cardiac remodeling with the formation of fibrotic and hypertrophic tissue seems to be the main contributor to the cardiomyopathy observed in patients [28,29,30]. The cardiac fibrotic being compromised can lead to myocardial infarction, heart failure and sudden death, resulting in poor outcomes [31,32].

Although there are many people at risk of infection and several patients at risk of developing heart fibrosis, there are still no effective treatments available which are focused on the damage caused by the progression of CD [33,34]. Therefore, advances in the search for new therapies for the cardiac manifestation are needed and in vitro models that offer some complexity similar to the microenvironment can favor this process. 

Our group was the first to characterize aspects of the *T. cruzi* infection in a spheroid model (also called microtissue). In 2008, Garzoni and collaborators were able to show, in a pioneer study, that the parasite invades and completes its cycle in 3D primary cardiac cell cultures, as observed in vivo. The results were demonstrated by transmission and scanning electron microscopy, as well as confocal microscopy [35].

Together with that, in this study, the reproduction of fibrosis and hypertrophy in these spheroids during infection was demonstrated for the first time [34]. In our cardiac fibrosis microtissue model we were able to observe the altered production of extracellular matrix proteins (ECM), inflammatory factors and enhanced volume. Also, it was shown to be susceptible to antifibrotic and trypanocidal treatments, such as SB431542, a selective inhibitor of TGF-β type I receptor, and Posaconazole, an antifungal that was being evaluated for CD treatment [36,37].

These findings provided new possibilities for CD studies in an in vitro system that is capable of reproducing heart tissue better than 2D cultures, and thus producing more clinically relevant results. Our primary 3D cardiac cell culture presents spontaneous contractility [Video S1] and is able to reproduce angiogenesis and vasculogenesis aspects, resembling the morphological and functional features of in vivo models [38]. From this, other groups have started to explore this model to investigate new therapeutic approaches to CD, such as trypanocidal and antifibrotic/anti-inflammatory compounds [39].

Thus, our primary cardiac spheroid model of fibrosis can be a great resource to fill the gaps that still exist in our understanding of CCC. It demonstrates similar aspects to the in vivo tissues, reproducing the parasite cell cycle and the main manifestations of cardiac CD in a controlled environment that is susceptible to drug response. As such, it can contribute to studies including infection mechanisms, novel therapies and molecular biomarkers that will add not only to new treatment strategies, but also to patients’ diagnoses [40]. In this sense, this review aims to explore the advantages of our 3D cardiac cell culture model of fibrosis in the context of CD, including fibrosis characterization, molecular targets and treatment aspects. This review is the first to gather studies using only 3D cultures from 2008 until 2024 that contribute to the CD cardiac fibrosis literature.

## 2. Methods Used to Analyze Primary Cardiac Microtissues in CD

In Chagas disease, several techniques can be applied for 3D cardiac culture analyses. However, we observed that Immunofluorescence, Western/Immunoblotting, Enzyme-linked immunosorbent assay (ELISA) and quantitative real-time PCR (qPCR) were the most used. Therefore, we aimed to gather the main applications of these methods from studies that use primary cardiac spheroids in the context of CD.

Using immunofluorescence, researchers were able to visualize extracellular matrix (ECM) protein expression during infection and after incubation with anti-fibrotic drugs. In addition, cardiomyocytes could be observed with Evans Blue staining and cellular and parasite DNA were labeled with DAPI [35,36,37]. To perform this technique, microtissues were initially fixed, permeabilized and incubated with the specific antibodies conjugated to a fluorophore. Thereby, the visualization of specific targets in different experimental conditions was allowed. 

Regarding Western/Immunoblotting, this is a method in which proteins must be extracted from a pool of spheroids and submitted to an electrophoresis separation. The separated proteins are transferred to an adsorbent membrane and incubated with antibodies that permit the detection, characterization and quantification of proteins of interest. It has been used in CD studies to quantify ECM proteins in the context of the infection and after trypanocidal and anti-fibrotic treatments [36,37]. 

ELISA is a technique that uses samples of the supernatants of the 3D cultures to detect and measure the necessary targets through antibody interaction. It allows for the analysis of the inflammatory environment of the disease, quantifying cytokines, such as tumor-growth factor (TGF-β), in the infected and treated microtissues [36,37].

Lastly, qPCR has been widely used to analyze parasite load in spheroids after infection and exposure to trypanocidal compounds. This technique is based on the extraction of total DNA from a pool of microtissues in different conditions. Subsequently, a primer is used to identify and amplify the targeted DNA. Thus, it is possible to quantify the parasite load and the amount of cells’ DNA separately [35,36,37].

## 3. The Use of Spheroids in Chagas Disease

Five articles were found in our research which addressed the use of the primary cardiac spheroids model to study cardiac fibrosis and related mechanisms in the context of CD (Table 1). The main topics approached included fibrosis characterization, molecular mechanisms and novel therapies.

### 3.1. Fibrosis Characterization

**Figure 1:**
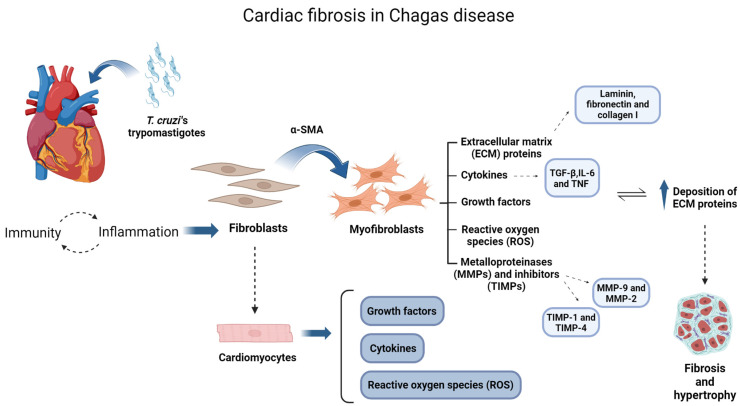
Schematic view of cardiac fibrosis mechanisms during *T. cruzi* infection. The invasion of the parasite in the cardiac tissue initiates inflammatory/immune responses, leading to activation of fibroblasts, the main effector cells in the process. Once activated, these cells express α-SMA and become myofibroblasts, which are able to secrete ECM proteins, cytokines and growth factors and modulate metalloproteinases (MMPs) and their inhibitors (TIMPs). Cardiomyocytes also contribute to this process, modulating cytokines, growth factors, and reactive oxygen species together with myofibroblasts. Therefore, with the persistence of the parasite in the heart tissue, a chronic inflammatory environment settles and the active phenotype of the fibroblasts remains in the tissue. Thus, the deposition of ECM continuously increases, leading to fibrotic and hypertrophic tissue. Cytokines, MMPs and ECM proteins demonstrated in the scheme are some of the targets already shown to be susceptible to antifibrotic/autoinflammatory treatments. Created using BioRender.com (agreement number PB26JRXTTA).

Fibrosis is one of the main characteristics of cardiac remodeling, together with hypertrophy [42]. As previously mentioned, it can be responsible for heart failure and the sudden death of patients with CCC [43]. The process of fibrotic tissue formation initiates with the inflammatory background, induced by the infection as illustrated in Figure 1. Cytokines and growth factors activate fibroblasts, the main cells responsible for the maintenance of the fibrotic tissue. In sequence, fibroblasts begin to produce large amounts of extracellular matrix (ECM) proteins, such as fibronectin, collagen and laminin. The parasite’s persistence in the tissues, together with the exacerbated inflammation, contributes to maintaining the active phenotype of these cells, promoting a progressive deposition of ECM proteins resulting in fibrotic tissue [44,45].

Four articles from our group addressed fibrosis characterization in the context of *T. cruzi* infection using primary cardiac spheroids. In 2008, Garzoni and collaborators showed, for the first time, that the infection of cardiac spheroids produced from mouse primary cell culture resulted in a four-fold increase in the area of the microtissues and a six-fold increase in its volume after 144 h, reproducing aspects of hypertrophy. Furthermore, this augmentation was related to the increased expression of extracellular proteins fibronectin, laminin and collagen IV [35]. These results indicated, for the first time, the reproduction of fibrotic characteristics in infected spheroids and were confirmed by the other three studies from the group [36,37,41]. Added to that, in 2013, Fares and collaborators presented new data showing that spheroids that were only exposed to the serum of patients with indeterminate (IND) and cardiac (CARD) CD manifestations also showed increased ECM proteins production, such as collagen I. Together with that, an increase in fibronectin expression when the cardiac spheroids were incubated with the serum of the IND group was observed. The laminin levels were not affected by serum exposure in any groups [41].

According to these findings, our primary 3D model successfully mimics the main clinical aspect of cardiac development after *T. cruzi* infection, fibrosis and hypertrophy. Beyond that, it is shown to be a potent model for studies about the impact of the immune response of the host in the cardiac tissue towards infection, making it a great tool for further evaluations of cardiac fibrosis in CD.

### 3.2. Molecular Mechanisms

Although many mechanisms remain to be uncovered in CD, it is already known that parasite persistence and inflammatory/immune/autoimmune responses are key factors in the development of cardiac disease [46]. The acute phase of the disease is marked by an intense inflammatory response, with mononuclear and polymorphonuclear cells, while the chronic phase is characterized predominantly by mononuclear cells [47,48]. Cytokines, such as tumor-growth factor (TGF-β), interferon-gamma (IFN-γ) and tumor necrosis factor (TNF) play an important role in CD cardiac pathogenesis [49,50]. Fibroblasts and cardiomyocytes, when activated by infection and inflammatory process, contribute to the modulation and increased production of these inflammatory cytokines, growth factors and reactive oxygen species [51,52]. In this way, the persistence of *T. cruzi* maintains the inflammation environment and the damage in heart tissues, leading to the progression of the Chagas cardiomyopathy [44,45] [Figure 1].

Thus, in our research, we found four articles approaching the use of our cardiac microtissue *T. cruzi*-infected model to study molecular mechanisms in the context of CD. Firstly, Fares and contributors, demonstrated in the 3D cardiac cultures that, when exposing these microtissues to serum of patients with IND CD, there was an increase in MMP-2 expression levels. Also, it was shown that incubation of the spheroids with the serum of CARD patients resulted in a tendency for the augmentation of MMP-9 levels. Thereby, it was observed for the first time the modulation of fibrotic factors in cardiac spheroids during the context of CD was observed for the first time [41]. Subsequently, in 2018, Ferrão and colleagues showed that a *T. cruzi* infection reduced the activity of MMP-2 and MMP-9 by 37% and 15%, respectively. Added to that, the data were able to show an increased production of TIMP-1 after infection, demonstrating the pro-fibrotic action of the parasite. In this way, the study presented new data showing that infection in the microtissues per se can also promote the modulation of factors engaged in the fibrosis formation process [36]. Further, another study observed that infected 3D cardiac spheroids had a 3.3-fold increase in α-SMA, a protein expressed in activated fibroblasts. Beyond that, the levels of ECM regulators, such as TGF-β and TIMP-4, were also augmented after infection, while MMP-9 levels were not affected. As such, the model again reproduced aspects of the inflammatory environment towards the infection, showing the participation of fibroblasts in this 3D system for the first time [37]. Lastly, it was demonstrated that the levels of inflammatory mediator IL-6 and TNF were increased after cardiac spheroid infection, confirming again the modulation of factors involved in cardiac pathogenesis. Concerning IL-10, IL-17, IL-4, IL-2 and IFN-γ, no change could be observed. Therefore, the data corroborated the reproducibility of the inflammatory prospect after infection, showing a broader participation of pro and antifibrotic factors in these infected cardiac microtissues [39]. 

Altogether, the data presented here exhibit that our in vitro microtissues are favorable models to study *T. cruzi*-related mechanisms, promoting new insights into the molecular targets in CD.

## 4. Three-Dimensional Cultures Applied for Drug Testing in Chagas Cardiomyopathy (CCC)

Usually, the first step in developing new therapies is in vitro testing. Regarding the currently used 2D cultures, they often bring results that cannot be reproduced in vivo. Nevertheless, in vivo systems are too complex to make specific analyses [53]. By mimicking the microenvironment of human organs more reliably, spheroids present more trustworthy drug test results, since their structure influences cell phenotype and response. In this sense, they permit the evaluation of pharmacokinetics and the prediction of efficacy and safety to be improved [54,55].

Regarding CD, the limitations of the therapeutic strategies available are some of the major challenges. As mentioned above, only two drugs are used in clinical settings for CD treatment, which were discovered in the 1970s. Benznidazole (BZ) and Nifurtimox (NF) are nitroimidazolic compounds with a trypanocidal effect, acting only on the parasite and not on the organ damage caused by the infection. Also, there are some restraints to their usage and they are mostly effective in the earlier phase of CD [56,57].

Currently available drugs for CCC are only palliative, such as β-blockers, angiotensin-converting enzyme inhibitors and antiarrhythmics, and do not act on the main manifestation, the heart fibrosis. Thus, therapies that focus on heart fibrosis are still needed [58,59].

### Disclosing New Treatments for Cardiac Fibrosis

As was mentioned previously, we are still lacking effective therapies for CD’s clinical manifestations [34,60]. This is especially important since in the acute phase most patients are asymptomatic or have generic symptoms, impairing earlier diagnosis and treatments [61]. As a result, patients become susceptible to severe cardiac developments, such as myocardial infarction and sudden death [62]. Because of this, the search for effective treatments that are aimed towards both parasites and the heart damage caused by disease progression is needed [63].

Therefore, two articles from our group approached drug testing to target fibrosis in 3D primary cardiac cultures infected with *T. cruzi*. In the first one, the treatment of infected cardiac spheroids with a selective inhibitor of TGF-β, a known cytokine that acts in the fibrosis process, reduced the size of the microtissues. Also, it was associated with a reduction in fibronectin expression promoted by the parasite, demonstrating for the first time action against fibrosis in our spheroids model [36]. Furthermore, in 2020, Nisimura et al. showed that the treatment of primary infected cardiac microtissues with Posaconazole, a drug with antifibrotic effects, reduced the production of fibronectin and laminin by 45% and 54%, respectively. These results corroborated previous findings, showing the susceptibility of the model to treatments, but also presented a new therapeutic possibility to cardiac fibrosis in the context of CD [37]. Thereby, our 3D primary cardiac culture not only reproduces clinical aspects of CD after infection but is also susceptible to treatments, being a great system to test new medications against the manifestations of the disease. 

## 5. Conclusions

In summary, 3D cell culture models present advantages regarding practicality, architecture and verisimilitude. In its microenvironment, molecular, physiological and pathogenic interactions happen in a more trustworthy way, mimicking the human body. Thereby, it offers opportunities for several research departments and enables the reproduction of a variety of organ systems. Beyond that, new applications are emerging for these microtissues, such as their use as building blocks to bioprinting, allowing for the construction of more complex models. CD is a disease with important cardiac involvement that largely benefits from these 3D systems. Our group has more than fifteen years of expertise in 3D primary cardiac cell cultures. Primary cardiac spheroids are capable of mimicking the contractility, angiogenesis, vasculogenesis and production of cellular factors such as ECM proteins and cytokines, resembling in vivo conditions. Thus, using this model, our group was the pioneer in developing a 3D primary cardiac cell culture that not only allows parasites to invade and complete their cycle, but also reproduces the main steps of fibrosis and hypertrophy formation in the heart tissue. After *T. cruzi* infection, these 3D primary cardiac cell culture models demonstrated a higher production of ECM proteins, enhanced volume of the microtissues, modulation of inflammatory cytokines and metalloproteases and was also susceptible to antifibrotic treatments. Therefore, the spheroids presented similar features to the heart tissue, including in the infection environment, contributing to more clinically relevant results. As such, it presented to be a great tool to elucidate some of *T. cruzi*’s fibrosis related physiopathological mechanisms, molecular targets and new therapy options. Finally, the data compiled in this paper support that our fibrosis 3D system is a great preclinical model to develop new insights about CD, providing perspectives that could benefit patients’ healthcare.

## Figures and Tables

**Table 1:** Research articles addressing the use of 3D primary cardiac cell cultures in Chagas disease included in this study.

Title	Autor	Year	Main Findings	Focus
Fibrosis and Hypertrophy Induced by *Trypanosoma* *cruzi* in a Three-Dimensional Cardiomyocyte- Culture System	Garzoni [35]	2008	In this new three-dimensional system, cardiac spheroids showed spontaneous contractility, with typical cardiac morphology and production of extracellular matrix components. There were four- and six-fold increases, respectively, in the area and the volume of *T. cruzi*–infected cardiomyocytes and whole microtissues, together with a 50% reduction in the cell population. Immunofluorescence showed the increased expression of fibronectin, collagen IV and laminin in the microtissues 144 h after infection. *T. cruzi* infection induced an increase in both the cellular area and the extracellular matrix components in cardiac spheroids, which contributed to an increase in the total microtissue volume, making this a powerful three-dimensional in vitro model for the study of cardiac-tissue hypertrophy, fibrosis, and remodeling.	- Fibrosis characterization
Matrix Metalloproteinases 2 and 9 Are Differentially Expressed in Patients with Indeterminate and Cardiac Clinical Forms of Chagas Disease	Fares [41]	2013	Using a new three-dimensional model of fibrosis, we observed that sera from patients with Chagas disease induced an increase in the extracellular matrix components in cardiac spheroids. Furthermore, MMP-2 and MMP-9 showed different correlations with matrix proteins and inflammatory cytokines in patients with Chagas disease. Our results suggest that MMP-2 and MMP-9 show distinct activities in Chagas disease pathogenesis. While MMP-9 seems to be involved in the inflammation and cardiac remodeling of Chagas disease, MMP-2 does not correlate with inflammatory molecules.	- Fibrosis characterization - Molecular mechanism
Inhibition of TGF-β pathway reverts extracellular matrix remodeling in *T.* *cruzi*-infected cardiac spheroids	Ferrão [36]	2018	Treatment with a selective inhibitor of TGF-β type I receptor, resulted in a reduction in the size of spheroids, and decreased parasite load and fibronectin expression as well as increased MMP-2 and a decrease TIMP-1 expression, which may be one of the mechanisms regulating extracellular matrix remodeling. The study discusses mechanisms by which the inhibition of TGF-β signaling reverses fibrosis and hypertrophy generated by *T. cruzi*.	- Molecular mechanisms - Novel therapies
Effect of Posaconazole in an in vitro model of cardiac fibrosis induced by *Trypanosoma cruzi*	Nisimura [37]	2020	Treatment with POS reduced parasite load by 50% according to real-time PCR and reduced fibrosis according to Western blot and immunofluorescence, which is associated with a reduction in fibronectin and laminin (45% and 54%, respectively). POS treatment also increased by 50% TGF-β and decreased by 58%; TIMP-4.	- Molecular mechanisms - Novel therapies
Benznidazole modulates release of inflammatory mediators by cardiac spheroids infected with *Trypanosoma cruzi*	Fiuza [39]	2021	BZ presented a low toxic profile on 3D matrices and a high potency in vitro according to a qPCR analysis of *T. cruzi*-infected cardiac spheroids. A flow cytometry appraisal of the inflammatory mediators released from the cellular supernatant showed increases in IL—6 and TNF in parasitized spheroids as compared to uninfected cultures. BZ at 10 μM suppressed the parasite load (92%) concomitantly decreasing in IL-6 (36%) and TNF (68%). Our findings corroborate the successful use of 3D cardiac matrices for in vitro identification of novel anti-parasitic agents and potential impact in host cell physiology.	- Molecular mechanisms

## Supplementary Materials

The following supporting information can be downloaded at: https://www.mdpi.com/article/10.3390/biomedicines12071410/s1. Video S1: Primary cardiac spheroids reproducing spontaneous contractility after seven days of culture, prior infection.
